# Isorhamnetin Exerts Antifibrotic Effects by Attenuating Platelet-Derived Growth Factor-BB-induced HSC-T6 Cells Activation via Suppressing PI3K-AKT Signaling Pathway

**DOI:** 10.61186/ibj.3948

**Published:** 2023-06-28

**Authors:** Mojtaba Rashidi, Emad Matour, Hasti Beheshti Nasab, Maryam Cheraghzadeh, Elham Shakerian

**Affiliations:** Cellular and Molecular Research Center, Medical Basic Science Research Institute, Department of Clinical Biochemistry, School of Medicine, Ahvaz Jundishapur University of Medical Sciences, Ahvaz, Iran

**Keywords:** Hepatitis, Liver injuries, PI3K-AKT

## Abstract

**Background:**

Currently, liver fibrosis is growing worldwide; unfortunately, there is no definite cure for this disease. Hence, understanding the molecular pathways involved in the development of liver fibrosis can help to find a proper treatment. In this study, we aimed to evaluate the effects of isorhamnetin as an antifibrotic agent on PDGF-BB-activated HSC-T6 cells in a concentration-dependent manner. We have also attempted to assess signaling pathways that may affect liver fibrosis.

**Methods:**

PDGF-BB was used to activate the HSC-T6 rat hepatic stellate cell line. The activated cells were treated with Isorhamnetin for 24 h. Finally, we compared the mRNA expression level of *COLA1* and *α-SMA* and also the level of phosphorylated *AKT* protein with the control group.

**Results:**

The obtained data revealed a significant increase in the expression level of the *COLA1* and *α-SMA* genes (p > 0.05), as well as phosphorylated *AKT* protein, in the cells treated with PDGF-BB. In addition, 75 and 100 µM concentrations of Isorhamnetin markedly declined the *COLA1* and *α-SMA *expression and also the phosphorylated *AKT* protein level in the HSC-T6 cells.

**Conclusion:**

Our findings suggest that Isorhamnetin decreases HSC-T6 activation, the expression of *COLA1* and *α-SMA*, in vitro, which could act as an antifibrotic element to reduce and treat liver fibrosis disease.

## INTRODUCTION

Today, liver fibrosis is a global health problem, which immunosuppressive factors or antiviral drugs inhibit its progression in the first stages. However, an efficient treatment is not available for patients with liver fibrosis, and a proper treatment is still far from satisfactory^[^^1^^,^^2^^]^. 

Excessive secretion of ECM and mass production of *COLA1* are the main signs of liver fibrosis onset and progression^[^^3^^,^^4^^]^. The primary origin of ECM is HSCs, which preserve keep retinoid and vitamin A and are inactive during physiological situations^[^^5^^,^^6^^]^. In hepatic fibrosis, activated HSCs express a large number of fibrogenic genes, including *COLA1* and *α-SMA*^[^^7^^]^. Indeed, HSCs are activated by various factors, undergo a transformation to a myofibroblast-like shape and express significantly higher levels of *COLA1* and *α-SMA*^[^^8^^]^. Besides, activated HSCs produce proliferative and fibrogenic elements that elevate both the proliferation rate of HSCs and the generation of ECM^[^^9^^]^. Preventing HSC activation and proliferation, inhibiting excessive ECM generation, and promoting ECM degradation are the main approaches for liver fibrosis treatment.

PDGF-BB is one of the most potential proliferative cytokines that activates HSCs. Following the liver injury, expression of PDGF-BB and its related receptor (PDGF-R) increases^[^^10^^]^. P13K/Akt is a signaling pathway, which controls the survival and growth of cells in response to extracellular signals. Previous studies have found that the PI3K/Akt signaling pathway has the ability to proliferate HSC, reduce HSC apoptosis, and regulate the liver fibrosis progression by degrading ECM and increasing the expression levels of *COLA1* and *α-SMA*^[^^11^^,^^12^^]^.

Isorhamnetin, a flavonoid isolated from the plant *Hippophae rhamnoides* L., has shown antioxidant, anti-inflammatory and antitumor activity^[^^13^^]^. It also exerts hepatoprotective effects by inhibiting the autophagy of hepatocytes and increasing apoptosis^[^^14^^]^. Nan et al.^[^^15^^]^ and Yang et al.^[^^16^^] ^have reported that isorhamnetin could weaken CCl4-induced hepatic fibrosis by reducing *TGF-β* mediated *Smad* signaling. However, it is unclear whether isorhamnetin can reduce hepatic fibrosis in the PDGF-BB-induced HSC-T6. The involvement of PI3K-AKT signaling pathway in the development of liver fibrosis has not yet been investigated, and there is currently little data on the mechanism of isorhamnetin action in liver fibrosis. The current study attempted to assess the effects of isorhamnetin treatment as an antifibrotic agents on PDGF-BB-activated HSC-T6 cells in a concentration-dependent manner. Also, the signaling pathways affecting liver fibrosis were evaluated.

## MATERIALS AND METHODS

**Chemicals and reagents**

PDGF-BB specific for cell culture, MTT, and DMEM, as well as penicillin and streptomycin antibiotics were all acquired from Sigma-Aldrich (Germany). FBS was provided from Gibco (USA).

**Cell culture conditions**

HSC-T6 is an immortalized cell line derived from male Sprague-Dawley retired breeder rats. It was obtained by transforming primary HSC using SV40 large T-antigen^[^^17^^]^. This cell line serves as a valuable model for studying metabolism due to its similarity to primary cells. HSC is the central effector in hepatic fibrosis^[^^4^^,^^18^^]^. The first passage of HSC-T6 cells was obtained from the Pasteur Institute of Iran (Tehran). The HSC-T6 cells were cultured in DMEM containing 10% FBS, 50 mg/ml of streptomycin, and 50 U/ml of penicillin^[^^19^^,^^20^^]^. To provide the optimal conditions for the cells, we changed the culture medium every 24 h until the cells reached 80% confluency. HSC-T6 cells were then kept in serum-starved FBS-free DMEM for 24 h and then exposed to 10 ng/ml of PDGF-BB.

**Effect of isorhamnetin on the viability of HSC-T6**

The MTT assay technique was employed to evaluate the cell viability^[^^21^^]^. The HSC-T6 cells without PDGF-BB treatment were cultured in a 96-well plate (density of 2 × 10^6^ cells/ml). After overnight incubation, the cells were treated with several concentrations of isorhamnetin (25, 50, 75, 100, 125, and 150 μM) for 24 h. Next, the supernatant of the cells was first removed, and then the cells were treated with isorhamnetin and incubated by MTT for 4 h. Finally, DMSO was used to solve the formazan crystals. ELISA plate reader was employed to read the absorbance at 570 nm.

**Treatment of** **PDGF-BB** **activated** **HSC-T6 cells** **with** **isorhamnetin**

To examine the inhibitory effects of isorhamnetin on the activation of PDGF-BB-induced HSC-T6 cells, different concentrations of isorhamnetin (25, 50, 75, 100, 125, and 150 μM) were used to treat the cells for 24 h. Real-time PCR was performed to evaluate the expression of genes involving in hepatic fibrosis, and Western blot analysis was employed to examine the levels of phosphorylated AKT in the cells activated with PDGF-BB and treated with the above-mentioned concentrations of isorhamnetin. The control group, without treatment, exhibited a similar condition to the treated groups in the laboratory.

**Measurement of gene expression using real-time PCR technique**

Following treatments, we used an RNA extraction kit (Qiagen, UK) to isolate the total RNA from the cells. Then the complementary DNA synthesis process was carried out by using oligo(dT) primers and random hexamer. We conducted real-time PCR by ABI Applied Biosystems (Thermo Fisher Scientific, USA) to have quantification of gene expression. The primers used in this study are listed in Table 1. GAPDH was also used as the internal control.

**Western blotting**

BCA Protein Assay Kit (Merck Millipore, Germany) was used to measure total protein concentrations. Samples were separated by using 10% Gel electrophoresis and then transferred to nitrocellulose membranes. Thereafter, the samples were incubated with both primary and secondary antibody, respectively. In the end, a ChemiDoc system (Bio-Rad, USA) was used to detect the intensity of the bands.

**Table 1:** Primer sequence for RT-PCR

**Gene**	**Primer sequence**	**PCR product (bp)**
*COLA1*	F: 5′-TGAAGGGACACAGAGGTTT-3′	188
	R: 5′-ACCATCATTTCCACGAGCA-3′	
		
*α-SMA*	F: 5′-TGGTGTCACCCACAATGTCC-3′	153
	R: 5′-ATCTCACGCTCAGCAGTAGT-3′	
		
*GAPDH*	F: 5′-CGGAGTCAACGGATTTGGTC-3′	181
	R: 5′-CTTCCCGTTCTCAGCCTTGA-3′	

** F: forward; R: reverse**

**Statistical analysis**

All the experiments were performed triplicate for reproducibility. One-way analysis of variance (ANOVA) was used to compare the significance of the differences among treatment groups. To define the significance of differences between the groups, multiple post hoc tests (Tukey's) was conducted. Results were statistically significant at p values of less than 0.05.

## RESULTS

**Impacts of isorhamnetin on the viability of HSC-T6 cells**

**Fig.1:**
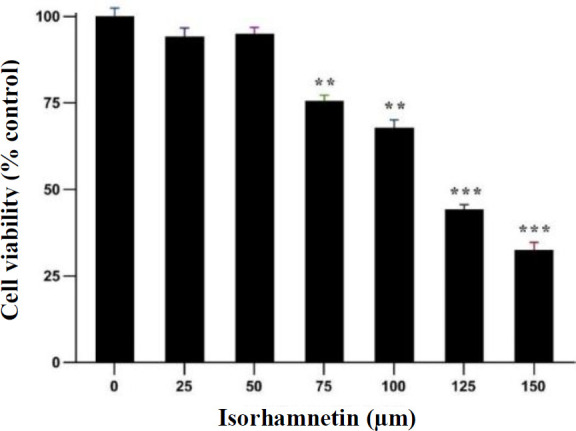
Isorhamnetin effects at different concentrations on the HSCs-T6 viability. The MTT assay data reveal the viability of the cells after 24 h. The results are presented with mean ± SEM. Analysis was done by Tukey test, one-way ANOVA, (^**^p < 0.01, ^***^p < 0.001).

The effects of several concentrations of isorhamnetin (25, 50, 75, 100, 125, and 150 μM) on the cell viability were evaluated using the MTT assay. Our findings revealed that concentrations exceeding 125 μM were toxic to the cell viability. Therefore, we decided to use lower concentrations of isorhamnetin in our experimental design to ensure the well-being of cells and validity of the results (Fig. 1).

**Impacts of Isorhamnetin on fibrogenic gene expression in PDGF-BB-treated HSC-T6 cells**

**Fig. 2:**
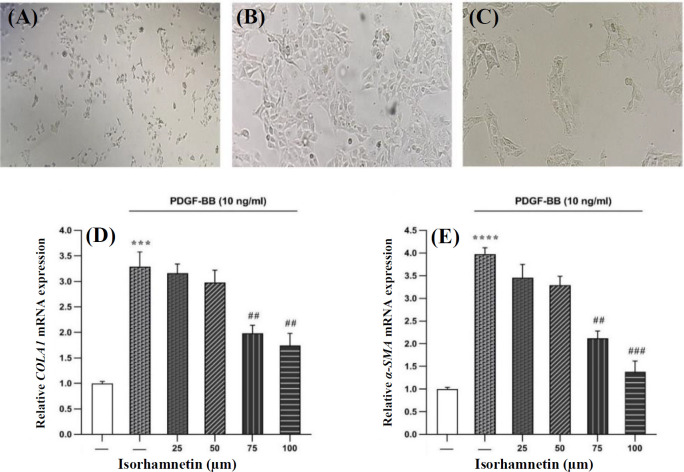
The cell shape of the control cells treated with PDGF-BB and isorhamnetin (A-C). The cells treated with PDGF-BB were activated, while isorhamnetin could inactive the cells. Effects of different concentrations of isorhamnetin on the *COLA1* (D) and *α-SMA* (E) gene expression in isorhamnetin-treated cells. The level of mRNA expression of *COLA1* and *α-SMA* genes reduced with 75 and 100 μM of isorhamnetin but did not reduce with 50 and 25 μM of this compound. Data are presented with the mean ± SEM and three replicates. GAPDH was the reference gene. (^***^p < 0.001 and ^****^p < 0.0001 vs. treated control; ^##^p < 0.01 and ^###^p < 0.001 vs. treated with isorhamnetin).

 Considering the phenotype and appearance of HSC-T6 cells, the transformation of quiescent HSC cells into fibrogenic myofibroblasts is the primary indicator of the activation of these cells. Activated cells had spindle-shaped, which were more elongated and converted to myofibroblasts, but inactive cells were virtually round ^[^^22^^,^^23^^]^. The shape of the control cells treated with PDGF-BB and isorhamnetin are shown in Fig. 2A- 2C. The cells treated with PDGF-BB were activated (Fig. 2B), while those treated with isorhamnetin were inactive (Fig. 2C). Modulation of *COLA1* gene expression was checked in the activated cells treated with 25, 50, 75, and 100 μM of isorhamnetin. RT-PCR results indicated a significant decline in the mRNA expression of *COLA1* (Fig. 2D) and *α-SMA* (Fig. 2E) in response to 75 and 100 μM of isorhamnetin compared to the cells treated only with PDGF-BB. The level of mRNA expression of *COLA1* and *α-SMA* genes reduced with 75 and 100 μM of isorhamnetin, but not with 50 and 25 μM of this compound. 

**Impact of isorhamnetin treatment on the PDGF-BB-induced phosphorylated AKT**

**Fig. 3:**
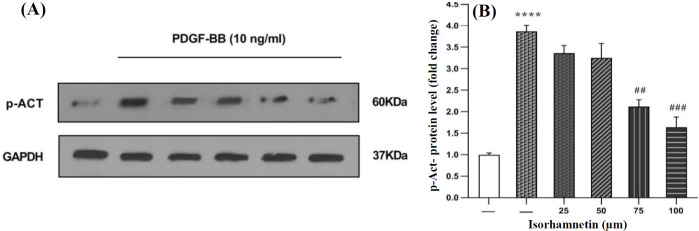
Analysis of PI3K-AKT signaling pathway by Western blotting (A). The cells were treated with 25, 50, 75, and 100 μM of isorhamnetin. (B) The relative PI3K-AKT level was expressed as the ratio PI3K-AKT/GAPDH. The data are presented with the mean ± SEM and three replicates (^****^p < 0.0001 vs. vehicle-treated control; ^##^p < 0. 01 and ^###^p < 0. 001vs. PDGF-BB alone)

Phosphorylated AKT was assessed to study the PI3K-AKT signaling pathway in the HSC-T6 cells treated with 75 and 100 μM of isorhamnetin and also to find out whether isorhamnetin could decline the PDGF-BB-induced PI3K-AKT signaling pathway to prevent hepatic fibrogenesis. Western blot data showed that 75 and 100 μM of isorhamnetin treatment significantly suppressed the phosphorylated *AKT* level compared to the PDGF-BB group (Fig. 3). These results showed the modulation of the PI3K-AKT signaling pathway by isorhamnetin.

## DISCUSSION

 This study investigates the effect of isorhamnetin treatment as an antifibrotic agent on the HSC-T6 cell line, which serves as a model for hepatic fibrosis. We studied whether isorhamnetin could attenuate the activation of HSC-T6 cells in vitro and also examined the influence of isorhamnetin on the expression of key genes implicated in the pathogenesis of liver fibrosis, namely *COLA1* and *α-SMA*, in vitro. We also examined the role of the PI3K-AKT signaling pathway in liver fibrosis.

Our findings suggested that the inhibitory action of isorhamnetin against hepatic fibrosis is through the reduction of HSC-T6 cell activation, which may be due to the effect of isorhamnetin on the PI3K-Akt signaling pathway. Although our understanding of the underlying mechanisms of hepatic fibrosis has significantly developed, definitive treatment for liver fibrosis remains elusive, posing a significant challenge in medicine. Our study provide evidence that isorhamnetin can inhibit the PI3K-Akt signaling pathway and suppress the expression of profibrogenic genes (*COLA1* and *α-SMA*) in PDGF-BB-induced HSC-T6 activation, indicating its potential as an antifibrotic agent in the HSC-T6 cell line. Excessive accumulation of ECM proteins, such as COLA1 and α-SMA, triggers liver fibrogenesis following the activation of HSCs. In a healthy liver, these cells remain inactive; however, following liver injury, the cells undergo a transformation, are activated and differentiated into myofibroblast cells that contribute to liver fibrosis^[^^24^^]^. 

Akt phosphorylation has been correlated with increased HSC replication, mRNA and COLA1 protein levels^[^^25^^]^. Previous research has shown that prevention of PI3-kinase, followed by *Akt* inhibition, through adenovirus-mediated transmission of a dominant-negative form of *Akt* significantly reduced HSC proliferation and elevation of COLA1 and α-SMA protein levels^[^^26^^]^. 

Several studies have identified PDGF-BB as a primary activator of genes involving in the development of liver fibrosis. Inhibition of these genes and attenuation of their associated signaling pathways may offer viable therapeutic strategies for liver fibrosis^ [^^1^^]^. Recent observations have demonstrated that flavonoid compounds may exert beneficial effects on liver fibrosis^[^^20^^]^. Wang and colleagues^ [^^27^^] ^have demonstrated that stimulation of HSCs by PDGF leads to cell proliferation and exacerbate fibrogenesis. In another study, Liu and colleagues^[^^28^^] ^examined the protective effects of isorhamnetin in preventing the progression of liver fibrosis induced by carbon tetrachloride (CCl_4_) in mice. They measured the expression of Smad3 and p38 mitogen-activated protein kinase proteins using Western blotting. Their results revealed that isorhamnetin inhibited HSC activation, ECM deposition, and autophagy. Isorhamnetin has a protective effect on liver fibrosis by reducing ECM production and inducing autophagy through the inhibition of the TGF-β1/Smad signaling pathway^[^^29^^]^. The potential to diminish HSC activation through the regulation of TGF-β1/Smads and PI3K/Akt signaling pathways suggests that these pathways could serve as valuable therapeutic targets for disease treatment^[^^30^^]^. 

Isorhamnetin demonstrated a significant reduction in the expression of key fibrogenic genes (*COLA1 *and *α-SMA*) in the activated HSC-T6 cells. The inhibitory effect of PDGF-BB-induced fibrosis is likely due to the PI3K-AKT signaling pathway inhibition. Therefore, isorhamnetin holds promise as a potential antifibrotic agent for mitigating liver injury, presenting a hopeful avenue for future research and potential therapeutic advancements.

## DECLARATIONS

### Ethical statement

The present study was designed and conducted with the approval of the Ethics Committee of Ahwaz Jundishapur University of Medical Sciences, Ahvaz, Iran (ethical code: IR.AJUMS.REC.1400.512).

### Data availability

The raw data supporting the conclusions of this article are available from the authors upon reasonable request.

### Author contributions

MR: designed the study and analyzed the data; EM: performed all assays and revised the manuscript; HBN: revised the manuscript and performed all assays; MC: analyzed the data; ES: designed the study, performed all assays, and wrote the first draft. All the authors have read and approved the final version of manuscript.

### Conflict of interest

None declared.

### Funding/support

This study was supported by the Ahvaz Jundishapur University of Medical Sciences (grant no. CMRC-0053). The funding source has no role in the design of the research, analysis, and interpretation of data, and publication of the manuscript.
